# Effects of Imatinib Mesylate (Gleevec) on Human Islet NF-kappaB Activation and Chemokine Production In Vitro

**DOI:** 10.1371/journal.pone.0024831

**Published:** 2011-09-14

**Authors:** Dariush Mokhtari, Tingting Li, Tao Lu, Nils Welsh

**Affiliations:** 1 Department of Medical Cell Biology, Uppsala University, Uppsala, Sweden; 2 Department of Molecular Genetics, Lerner Research Institute, Cleveland Clinic Foundation, Cleveland, Ohio, United States of America; French National Centre for Scientific Research, France

## Abstract

**Purpose:**

Imatinib Mesylate (Gleevec) is a drug that potently counteracts diabetes both in humans and in animal models for human diabetes. We have previously reported that this compound in human pancreatic islets stimulates NF-κB signaling and islet cell survival. The aim of this study was to investigate control of NF-κB post-translational modifications exerted by Imatinib and whether any such effects are associated with altered islet gene expression and chemokine production in vitro.

**Procedures:**

Human islets were either left untreated or treated with Imatinib for different timepoints. IκB-α and NF-κB p65 phosphorylation and methylation were assessed by immunoblot analysis. Islet gene expression was assessed using a commercial Pathway Finder microarray kit and RT-PCR. Islet chemokine production was determined by flow cytometric bead array analysis.

**Findings:**

Human islet IκB-α and Ser276-p65 phosphorylation were increased by a 20 minute Imatinib exposure. Methylation of p65 at position Lys221 was increased after 60 min of Imatinib exposure and persisted for 3 hours. Microarray analysis of islets exposed to Imatinib for 4 hours revealed increased expression of the inflammatory genes IL-4R, TCF5, DR5, I-TRAF, I-CAM, HSP27 and IL-8. The islet release of IL-8 was augmented in islets cultured over night in the presence of Imatinib. Following 30 hours of Imatinib exposure, the cytokine-induced IκB-α and STAT1 phosphorylation was abolished and diminished, respectively. The cytokine-induced release of the chemokines MIG and IP10 was lower in islets exposed to Imatinib for 30 hours.

**Conclusion:**

Imatinib by itself promotes a modest activation of NF-κB. However, a prolonged exposure of human islets to Imatinib is associated with a dampened response to cytokines. It is possible that Imatinib induces NF-κB preconditioning of islet cells leading to lowered cytokine sensitivity and a mitigated islet inflammation.

## Introduction

Type 1 diabetes is an autoimmune disease in which dysfunction and damage of insulin-producing beta-cells is thought to arise from direct contact with immune cells and from exposure to cytotoxic pro-inflammatory cytokines and other toxic substances [1]. In Type 2 diabetes beta-cells are also dysfunctional and damaged, possibly in response to peripheral insulin resistance, hyperglycemia, hyperlipidemia and cytokines, leading to a relative lack of insulin [2]. The molecular events leading to diabetes-associated beta-cell dysfunction and death have been investigated and it appears that the activation of the transcription factor Nuclear factor kappa B (NF-κB), in response to both inflammation, amyloidogenic peptides and oxidative stress, plays a central role in this chain of events [1].

The transcription factor NF-κB is activated by different mitogenic stimuli, stress signals, or inflammatory cytokines in a broad range of cell types [3], [4]. The classical NF-κB pathway involves the NF-κB precursor protein p105 (NF-κB-1) [5]. This protein is processed to the mature p50 NF-κB-1 that preferentially heterodimerizes with other members of the NF-κB family, i.e. p65, c-Rel or Rel-B. Activation of the classical pathway involves release of p50/p65 from IκB-α, as a result of phosphorylation by the IκB kinase and degradation of IκB-α by the proteasome. The mature dimeric NF-κB proteins then translocate to the nucleus and activate genes involved in anti-apoptotic function, the modulation of immune and inflammatory response, cell proliferation, adhesion, and angiogenesis [6].

The drug Imatinib mesylate (Gleevec) is currently used as treatment of chronic myeloid leukemia (CML), Gastrointestinal stromal tumor (GIST) and other malignancies, diseases caused by the Bcr-Abl oncogene, c-Kit mutations or other tyrosine kinase mutations. Imatinib binds to and stabilizes the inactive form of Bcr-Abl, which leads to annulation of the effects of the Bcr-Abl oncoprotein through the inhibition of Bcr-Abl autophosphorylation and substrate phosphorylation [7]. Imatinib is the first member of a new class of agents that act by specifically inhibiting a certain mutated enzyme that is characteristic of a particular cancer cell, rather than non-specifically inhibiting and killing all rapidly dividing cells, and it reached clinical practice some 10 years ago.

It has recently been observed that patients suffering from both leukemia and diabetes were cured from not only leukemia, but also diabetes, when treated with Imatinib [8]–[10]. Although two subsequent studies found no effect of Imatinib on Type 2 diabetes [11], [12], other additional studies report anti-hyperglycemic/anti-diabetic effects of Imatinib or similar tyrosine kinase inhibitors in humans [13]–[20]. These clinical studies clearly demonstrate that although not all Type 2 diabetes patients benefit from Imatinib therapy, there is a substantial proportion that responds, and some cases so dramatically that they become insulin-independent. An anti-diabetic action of Imatinib in Type 2 diabetes is further supported by our recent observation that Imatinib counteracts high-fat diet induced insulin resistance and hyperglycemia in rats [21]. Moreover, in a study from 2009, Imatinib was also observed to induce remission of diabetes in *db/db* mice, possibly via decreasing insulin resistance and increasing the beta-cell mass [22]. Also, Imatinib has been demonstrated to counteract streptozotocin-induced diabetes in rats [23]. Thus, in both animal models and in Type 2 diabetes patients Imatinib seems to improve glycemic control, possibly via an insulin sensitizing effect.

Imatinib appears to prevent and reverse not only Type 2 diabetes, but also diabetes of animal models with a Type 1 diabetes resembling disease. We have shown that Imatinib protects against beta-cell death *in vitro* and prevents diabetes in NOD mice and in streptozotocin-diabetic mice, both models for human beta-cell destruction and Type 1 diabetes [24], [25]. More recently, it has been observed that both Imatinib and Sunitinib not only prevented, but also reversed new-onset diabetes in NOD mice [26]. Thus, there exists proof-of-principle in animal models for an anti-diabetic effect of Imatinib and similar tyrosine kinase inhibitors, and that a limited treatment period will not only reverse diabetes, but also mediate long-term protection against re-precipitation of the disease [26]. This has led us [27] and other investigators [28], [29] to propose clinical trials in which Imatinib is given to new-onset Type 1 diabetes patients.

We have previously reported that Imatinib-mediated protection against human islet cell death was paralleled by increased IκB degradation [25]. We have also observed that increased NF-κB activity in human islet cells resulted in enhanced survival in response to different beta-cell toxins [30]. Thus, Imatinib may promote beta-cell survival by increasing expression of NF-κB induced anti-apoptotic genes. The effects of Imatinib on human islet inflammation are, to our knowledge, unknown. Therefore, we presently investigated human islet IκB/NF-κB phosphorylation, acetylation and methylation, and studied whether islet gene expression and chemokine production was affected by Imatinib treatment. We report that Imatinib promoted a modest and rapid increase in IκB phosphorylation, p65 phosphorylation and p65 methylation. Imatinib also enhanced expression of NF-κB responsive inflammatory genes and increased production of the chemokine IL-8. We also observe that a prolonged Imatinib exposure preconditioned the islet cells so that the cytokine response, as assessed by both IκB and STAT1 phosphorylation and chemokine production, was attenuated. We propose that the Imatinib-mediated increase in NF-κB results in human islet desensitization to cytokines and decreased islet inflammation. These finding may improve our understanding of the mechanisms by which Imatinib counteracts both Type 1 and Type 2 diabetes.

## Materials and Methods

### Islet isolation, culture, and exposure to different beta-cell inhibitory substances

Human pancreatic islets were kindly provided by Prof. Olle Korsgren (Dept. of Radiology, Oncology and Clinical Immunology, Uppsala University Hospital, Uppsala, Sweden) through the Nordic Network for Clinical Islet Transplantation. Only organ donors who had agreed to donate for scientific purposes were included. Informed written consent to donate organs for medical and research purposes was obtained from donors, or relatives of donors, by the National Board of Health and Welfare (Socialstyrelsen), Sweden. Permission to obtain pancreatic islet tissue from the Nordic Network for Clinical Islet Transplantation was reviewed and approved by the local ethics committee (Regionala etikprövningsnämnden, Uppsala) in Uppsala, Sweden. The human pancreatic islets were isolated from the pancreas of brain-dead organ donors using collagenase digestion and Biocoll gradient centrifugation [31].

After isolation, the islets were cultured free-floating in Sterilin dishes in CMRL 1066 medium containing 5.6 mM glucose, 10% fetal calf serum (FCS), and 2 mM L-glutamine for 1–5 days.

The islet beta-cell percentages determined using Newport Green staining followed by fluorescence microscopy were routinely 30–60%. To evaluate islet functional quality, batches of twenty islets were perfused with Krebs-Ringer bicarbonate HEPES buffer (KRBH) containing 2 mg/ml human albumin and 1.67 mmol/l glucose at a flow rate of 0.3 ml/min. The glucose concentration was increased to 16.7 mM after a 30-min period. Fractions for insulin measurement were collected every 4 min. Only islet preparations that responded to a high glucose challenge with a more than doubled insulin release were used for further experimentation.

Following the initial culture period, islets were cultured for an additional 6–24 hours in CMRL 1066 containing antibiotics, 2 mM glutamine and one of the following supplements: 0.5 mM sodium palmitate solubilized in 0.5% (weight/volume) fatty acid and lipopolysaccaride free bovine serum albumin (BSA) (Sigma); recombinant human Interleukin 1beta (IL-1ß (50 units/ml) (PeproTech, London, U.K.) and Interferon-gamma (IFN-γ) (1,000 units/ml) (PeproTech); 100 mM hydrogen peroxide; 2 mM DETA/NO (Cayman Chemicals) or 10 mM streptozotocin (STZ) (Sigma). To some of the groups 10 µM of Imatinib was added at different time points prior to the addition of test substances given above. To controls equal amounts of vehicle (DMSO) were supplemented.

### RNA isolation and cDNA synthesis

Total RNA was isolated from human pancreatic islet using the Ultraspec™ RNA Isolation System reagent (Biotecx Laboratories, Houston, TX, USA). Two µg of RNA was used for cDNA synthesis. cDNA was synthesized using the M-MuLV reverse transcriptase (Finnzymes, Espoo, Finland) and oligo-dT primers according to the manufacturer's instructions.

### Real-time PCR

PCR amplification was performed using the Lightcycler instrument (Roche Diagnostics, Lewes, UK) and the Lightcycler FastStart DNA Master SYBR Green I kit (Roche Diagnostics). The following primers were used: GAPDH (human) (glyceraldehyde-3-phosphate dehydrogenase): 5′-ACCACAGTCCATGCCATCAC (forward) 5′-TCCACCACCCTGTTGCTGTA (reverse), IL-8 (human) 5′-GGCAACCCTACAACAGACCCACA (forward), TGCTAACAGGCTTAGGACTTGC (reverse). Semi-quantitative data for IL-8 expression were calculated using the formula: 2^crossing point GAPDH - crossing point IL-8^.

### Immunoblot analysis

Islets in groups of 50 were washed once with PBS and then lysed with SDS sample buffer; 2% SDS, 5% β-mercaptoethanol, 100 mM Tris-HCL pH 6.8, 10% glycerol and bromophenolblue. The protein samples were boiled for 5 min before separation on a 9% SDS-PAGE. The separated proteins were electrophoretically transferred to Hybond™-P membranes (Amersham Bioscience), which were then pre-blocked in 5% BSA. The membranes were hybridized with monoclonal mouse or polyclonal rabbit antibodies directed against IκB-α (C-21, Santa Cruz), Phospho-Ser32- IκB-α (Cell Signaling), Acetylated-Lys310-p65 (Abcam), Phospho-Ser276-p65 (Santa Cruz), Phospho-Ser536-p65 (Cell Signaling), p65 (F-6, Santa Cruz), Methyl-Lys221-p65 [32] or Phospho-Tyr701-STAT1 (Cell Signaling) over night at 4°C. The horseradish peroxidase conjugated anti-mouse or anti-rabbit antibodies were used as secondary antibodies and luminescence detected with the ECL system (Amersham Bioscience). The resulting bands were then digitalized using the Kodak 4000MM system and the optical densities were measured using the Kodak Molecular Imaging Software.

### Extraction of nuclear proteins and electromobility shift assay (EMSA)

INS-1 832/13 cells were treated with Imatinib (10 µM) for 1 or 24 hours and IL-1ß (50 U/ml)+IFN-γ (1000 U/ml) for 30 min, after which nuclear proteins were extracted for EMSA, which was performed as previously described [32]. The κB-binding activity of the nuclear extracts was determined using the LightShift Chemiluminescent EMSA Kit (Pierce Biotechnology) according to the instructions of the supplier. As labeled probe, a double-stranded biotin-labeled oligonucleotide with the sequence 5′-AGTTGAGGGGACTTTCCCAGGC was used. The resulting bands were digitalized using the Kodak 4000MM system and the optical densities were measured using the Kodak Molecular Imaging Software.

### Microarray analysis

Human islets in groups of 1000 were either treated with DMSO or with 10 µM Imatinib for 4 hours. Following the treatments, total RNA was isolated using the Ultraspec™ RNA Isolation System reagent (Biotecx Laboratories, Houston, TX, USA). The RNA was then converted to biotin-labelled cRNA using the TrueLabeling-AMP™ Linear RNA Amplification Kit (SABiosciences) and hybridized overnight to the the Oligo GEArray Human Signal Transduction Pathway Finder Microarray filters (SABiosciences). Signals from the filters were detected by luminescence and quantified by densitometry using the Kodak 4000MM system and Kodak Molecular Imaging Software, all according to the to the instructions of the supplier (SABiosciences).

### Chemokine analysis

Human islets in groups of 50 were cultured in 0,5 ml CMRL 1066. The islets were pre-cultured for 6 hours with our without 10 µM of Imatinib and then cultured for another 24 hours with either 100 µM hydrogen peroxide, 10 mM streptozotocin, 1 mM DETA/NO or cytokines (50 U/ml IL-1ß+1000 U/ml IFN-γ). Islet culture medium was then recovered and analyzed for interleukin-8 (CXCL8/IL-8), RANTES (CCL5/RANTES), monokine induced by interferon-γ (CXCL9/MIG), monocyte chemoattractant protein-1 (CCL2/MCP-1), and interferon-γ–induced protein-10 (CXCL10/IP-10) levels using the BD Cytometric Bead Array Human Chemokine Kit according to the instructions of the supplier (Becton Dickinson).

## Results

### Imatinib induces a rapid increase in IκB-α phosphorylation, ser276-p65 phosphorylation and lys221-p65 methylation

**Figure 1:**
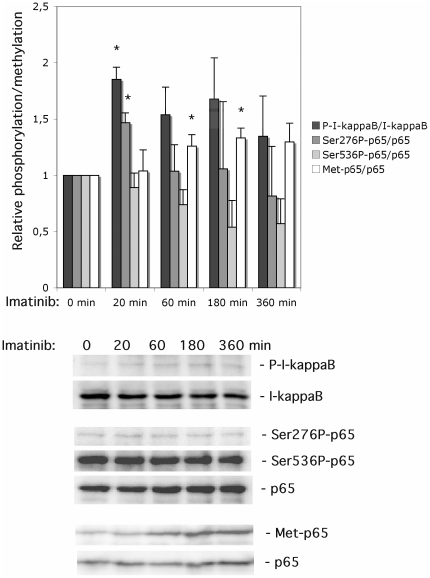
Effects of Imatinib on human islet IκB-α and NF-κB p65 phosphorylation/methylation. Human islets in groups of 50 were cultured in the presence of vehicle (DMSO) or 10 µM of Imatinib at the times given in the Figure. Islets were washed and harvested for SDS-PAGE and immunoblotting with anti-IκB-α and p65 antibodies. Phospho- and methylation-bands were quantified and normalized to total IκB-α and p65 bands. The lower panel shows immunoreactivity from one representative experiment. Results are means ± SEM for 4 independent experiments. * denotes p<0.05 using paired Student's t-test when comparing vs. control.

We have previously reported that Imatinib promoted IκB-α degradation in human islets [25]. To better understand the effects of Imatinib on human islet NF-κB activation, we first performed time course studies of IκB-α phosphorylation, Ser276-p65 phosphorylation, Ser536-p65 phosphorylation, Lys310-p65 acetylation and Lys221-p65 methylation. These are all NF-κB-associated post-translational modifications that have been reported to stimulate NF-κB activity [33]–[36]. We observed that IκB-α phosphorylation was significantly increased at 20 min of Imatinib (10 µM) exposure (Figure 1). At later time points there was a trend to increased IκB-α phosphorylation, but it did not reach statistical significance, probably due to the low number of observations. Ser276-phosphorylated p65 migrated slightly slower (72 kDa) than non-ser276-phosphorylated p65 (68 kDa), and the intensity of the P-ser276-p65 band was weak. This indicates that P-ser276-p65 constitutes a minor proportion of all p65 and this fraction could represent the transcriptionally active form of p65. The phosphorylation of p65 at position Ser276 was increased by Imatinib at 20 min (Figure 1). At 60–360 min, however, the ser276-p65 phosphorylation was no longer increased, indicating that Imatinib-induced ser276-p65 phosphorylation is transient. P-Ser536-p65 immunoblotting signals were strong and P-ser536-p65 migrated to the same position as total p65. This finding is consistent with the view that p65 is constitutively phosphorylated at position ser536 in human islets, and that this post-translational modification has limited regulatory importance. Indeed, Imatinib did not stimulate ser536-p65 phosphorylation; instead there was a trend towards decreased ser536-p65 phosphorylation with increasing exposure time to Imatinib (Figure 1). Methylation of p65 as position lys221 was not affected by Imatinib at 20 min. However, at both 60 and 180 min Imatinib promoted a modest increase in p65 methylation (Figure 1), which was less pronounced than that of cytokines (results not shown). Finally, we probed for effects of Imatinib on p65 Lys310 acetylation. However, we could not detect specific immunreactivity for Ac-lys310-p65 in human islets, neither under conditions of Imatinib exposure or when stimulated by cytokines (results not shown). It is not clear whether the lack of Ac-p65 signal is due to a low sensitivity of the Abcam anti-Ac-p65 antibody, or to a constitutively low p65 acetylation activity in human islets.

### Four hours of Imatinib exposure results in increased expression of inflammatory genes

To verify that the observed effects of Imatinib on human islet IκB-α and p65 phosphorylation/methylation events result in increased NF-κB target gene transcription, we performed a microarray analysis. Human islets were exposed to vehicle or Imatinib for 4 hours and then analyzed using the Oligo GEArray Human Signal Transduction Pathway Finder Microarray. By this approach we observed that 10 genes were increased more than 30% in response to the Imatinib treatment (Table 1). Out of these 10 genes 7 are typical inflammatory or stress genes (IL-4R, TCF5, DR5, I-TRAF, I-CAM, HSP27 and IL-8), indicating that Imatinib promoted transcription of NF-κB target genes.

**Table 1:** Effects of imatinib on human islet gene expression as assessed by microarray analysis.

GeneBank	Description	Gene Name	Fold induction (Imatinib vs control)
NM_000418	Interleukin 4 receptor	IL-4 Ra	1.64
NM_005526	Heat shock transcription factor 1	Tcf5	1.48
NM_003842	Tumor necrosis factor receptor superfamily, member 10b	KILLER/DR5/TRAILR2	1.40
NM_004180	TRAF family member-associated NF-κB activator	I-TRAF	1.39
NM_000201	Intercellular adhesion molecule 1 (cd54), human rhinovirus receptor	ICAM-1	1.36
NM_002539	Ornithine decarboxylase 1	ODC1	1.34
NM_001540	Heat shock 27 kDa protein 1	HSP28/HSP27/Hsp25	1.33
NM_000584	Interleukin 8	IL-8	1.32
NM_005967	NGFI-A binding protein 2 (DGR1 binding protein 2)	NAB2	1.32
NM_001552	Insulin-like growth factor binding protein 4	IGFBP-4	1.31

Human islets in groups of 1000 were exposed to DMSO (control) or to 10 µM of Imatinib for 4 hours. Following treatments, RNA was isolated, converted into cRNA and hybridized to Oligo GEArray Human Signal Transduction Pathway Finder Microarray filter according to the instructions of the supplier. The filters were scanned in a Kodak 4000MM Image station. Results from two independent experiments were analyzed by measuring the optical densities of the spots using the Kodak Molecular Imaging Software. Optical densities were normalized to housekeeping genes included on the filter (ribosomal protein S27a, GAPDH, beta-actin). Table shows the mean fold induction from the two separate experiments and only genes increased with more than 30% are shown.

### Six hours of Imatinib exposure does not affect cytokine-induced IκB-α and ser276-p65 phosphorylation

**Figure 2:**
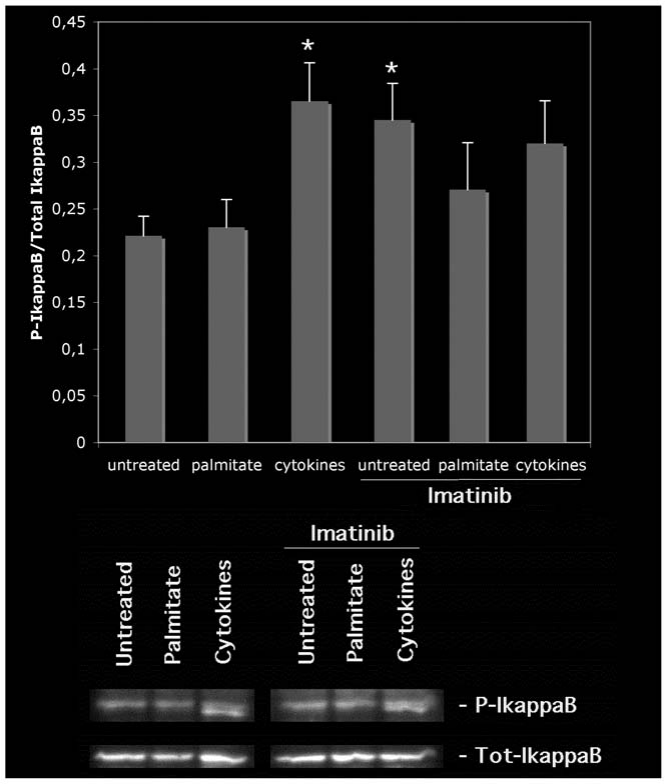
Effects of cytokines, palmitate and Imatinib on human islet IκB-α phosphorylation. Human islets in groups of 50 were pre-cultured for 6 hours with our without 10 µM of Imatinib and then stimulated with either 0.5 mM sodium palmitate or 50 U/ml IL-1β+1000 U/ml IFN-γ for 20 min. Islets were washed and harvested for SDS-PAGE and immunoblotting with anti-P-IκB-α and total IκB-α antibodies. Phospho-bands were quantified and normalized to total IκB-α bands. The lower panel shows immunoreactivity from one representative experiment. Results are means ± SEM for 4 independent experiments. * denotes p<0.05 using paired Student's t-test when comparing vs. control.

**Figure 3:**
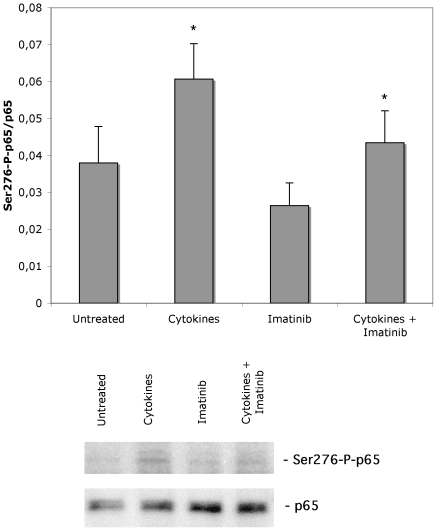
Effects of cytokines and Imatinib on human islet NF-κB p65 phosphorylation. Human islets in groups of 50 were pre-cultured for 6 hours with our without 10 µM of Imatinib and then stimulated with 50 U/ml IL-1β+1000 U/ml IFN-γ for 20 min. Islets were washed and harvested for SDS-PAGE and immunoblotting with anti-P-Ser276-p65 and total p65 antibodies. Phospho-bands were quantified and normalized to total p65 bands. The lower panel shows immunoreactivity from one representative experiment. Results are means ± SEM for 3 independent experiments. * denotes p<0.05 using paired Student's t-test when comparing vs. control.

Because IκB-α and ser276-p65 phosphorylation were stimulated by Imatinib (Figure 1), we next investigated whether cytokine-induced IκB-α and ser276-p65 phosphorylation was affected by Imatinib treatment. We also studied the effects of sodium palmitate, since it has been reported that this saturated free fatty acid promotes NF-κB activation in human beta-cells [37]. We observed that a 20 min exposure to the combination of IL-1ß (50 U/ml) and IFN-γ (1000 U/ml) increased IκB-α phosphorylation (Figure 2). Somewhat surprisingly, sodium palmitate was without effect (Figure 2). Similar to the results of Figure 1, a 6-hour Imatinib pre-exposure promoted an increased IκB-α phosphorylation. Interestingly, cytokine-induced IκB-α phosphorylation in the presence of Imatinib was not further increased as compared to Imatinib only (Figure 2). Using the same experimental setup as in Figure 2, we observed that cytokines also promote phosphorylation of ser276-p65 (Figure 3). Cytokine-induced ser276-p65 phosphorylation was not affected by the 6-hour Imatinib pretreatment (Figure 3). Thus, at the 6-hour time point, cytokine-induced IκB-α and ser276-p65 phosphorylation is not further potentiated by Imatinib.

### Over-night exposure to Imatinib results in attenuation of cytokine-induced IκB-α and STAT1 phosphorylation

**Figure 4:**
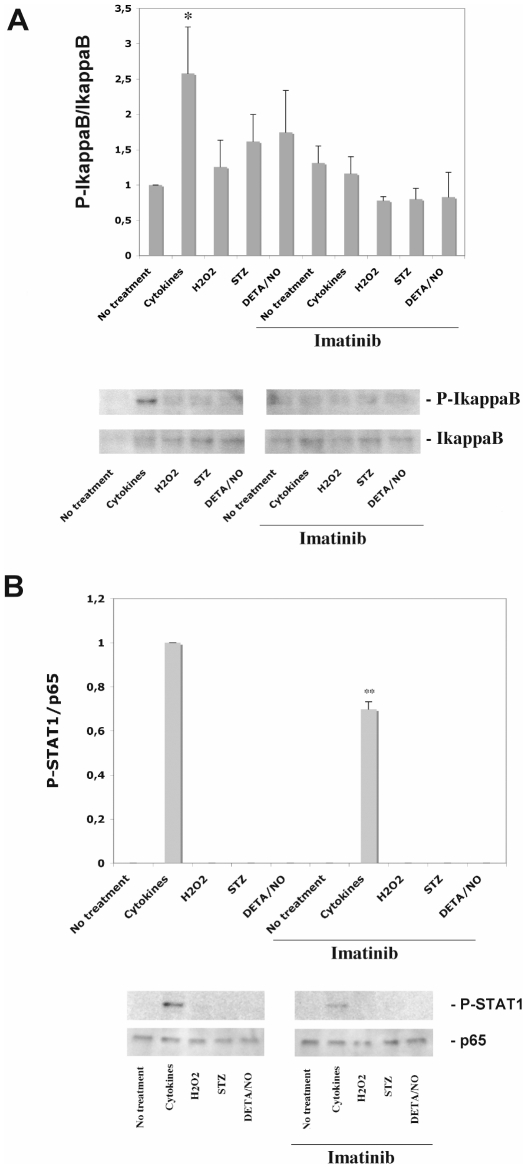
Effects of cytokines, hydrogen peroxide, STZ, DETA/NO and Imatinib on human islet IκB-α (A) and STAT1 (B) phosphorylation. Human islets in groups of 50 were pre-cultured for 6 hours with our without 10 µM of Imatinib and then cultured for another 24 hours with either 50 U/ml IL-1β+1000 U/ml IFN-γ (cytokines), 100 µM hydrogen peroxide (H_2_O_2_), 10 mM streptozotocin (STZ) or 1 mM DETA/NO. Islets were washed and harvested for SDS-PAGE and immunoblotting with anti-P-IκB-α and total IκB-α antibodies (A) or anti-P-STAT1 and total p65 antibodies (B). Phospho-bands were quantified and normalized to total IκB-α or total p65 bands. The lower panels show immunoreactivity from one representative experiment. Results are means ± SEM for 3 independent experiments. * denotes p<0.05 using paired Student's t-test when comparing vs. control.

To clarify whether a prolonged (over-night) Imatinib-exposure period affects cytokine-induced signaling, we pre-exposed human islets to Imatinib for 6 hours and then continued the culture period for another 24 hours with and without both Imatinib and the combination of IL-1ß and IFN-γ. In this situation human islet IκB-α phosphorylation was enhanced in cells exposed to cytokines, but not in cells exposed to both cytokines and Imatinib (Figure 4A). Interestingly, also cytokine-induced STAT1 phosphorylation was weakened by Imatinib (Figure 4B). Thus, a prolonged Imatinib exposure resulted in attenuation of cytokine-induced signaling events.

We also tested whether human islet IκB-α phosphorylation was affected by the beta-cell toxins hydrogen peroxide, which induces oxidative stress, streptozotocin, which induces alkylation of macromolecules and NAD+ depletion, and DETA/NO, which induces nitrosative stress. As we have previously observed that beta-cell death induced by these toxins is counteracted by Imatinib [24], [25], it could be hypothesized that stressed beta-cells activate NF-κB and that this event is modulated by Imatinib. Although these substances tended to increase IκB-α phosphorylation, there were no significant effects, either induced by themselves or in combination with Imatinib (Figure 4A). Moreover, STAT1 phosphorylation was undetectable in all groups other than the cytokine-treated islets (Figure 4B). The limited access of human islet material precludes a more sensitive and careful analysis of these parameters, something that is probably necessary to successfully detect modest alterations in NF-κB and STAT1 signaling.

### Imatinib increases basal and decreases cytokine-induced chemokine release

**Figure 5:**
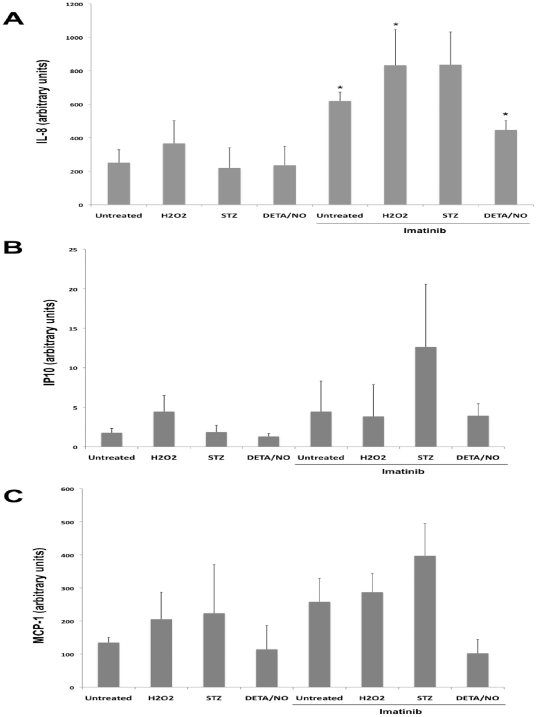
Effects of hydrogen peroxide, STZ, DETA/NO and Imatinib on human islet IL-8 (A), IP10 (B) and MCP-1 (C) release. Human islets in groups of 50 were pre-cultured for 6 hours with our without 10 µM of Imatinib and then cultured for another 24 hours with either 100 µM hydrogen peroxide (H_2_O_2_), 10 mM streptozotocin (STZ) or 1 mM DETA/NO. Islet culture medium was then recovered and analyzed for IL-8 (A), IP10 (B) and MCP-1 (C) contents using a flow cytometry cytometric bead assay. Results are expressed in arbitrary units and are means ± SEM for 3 independent experiments. * denotes p<0.05 using paired Student's t-test when comparing vs. control.

**Figure 6:**
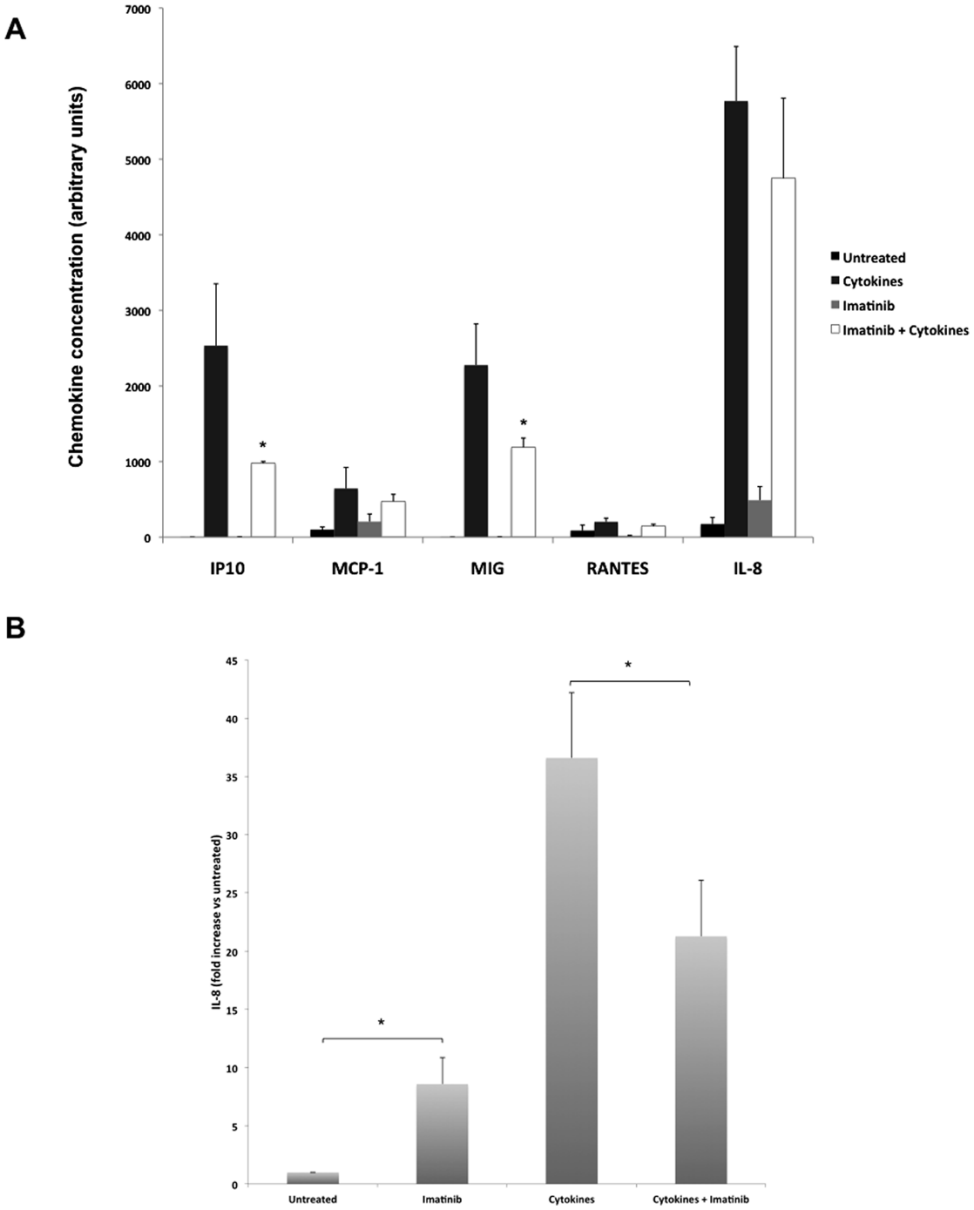
Effects of cytokines and Imatinib on human islet chemokine release and on IL-8 mRNA levels. **A.** Human islets in groups of 50 were pre-cultured for 6 hours with our without 10 µM of Imatinib and then cultured for another 24 hours with 50 U/ml IL-1β+1000 U/ml IFN-γ (cytokines). Islet culture medium was then recovered and analyzed for the chemokines IP10 and MCP-1, MIG, RANTES and IL-8 contents using a flow cytometry cytometric bead assay. Results are expressed in arbitrary units and are means ± SEM for 3 independent experiments. * denotes p<0.05 using paired Student's t-test when comparing vs. control. **B.** Human islets in groups of 30 were left untreated or treated with Imatinib (10 µM, 30 hours) or cytokines (50 U/ml IL-1β+1000 U/ml IFN-γ, 24 hours). In the Imatinib+cytokines group, islets were pretreated with Imatinib for 6 hours followed by a 24 hours exposure to cytokines. Following treatments, RNA was isolated and cDNA synthesized and IL-8 levels analyzed using real time PCR. Figure shows fold induction when normalized to GAPDH. Results are means ± SEM for 4 independent experiments. * denotes p<0.05 using paired Student's t-test when comparing vs. control.

To obtain a functional read-out of Imatinib-modulated NF-κB signaling events, we analyzed human islet chemokine release using a flow cytometric bead assay for the chemokines IP10, IL-8, MCP-1, RANTES and MIG. The experimental setup was the same as for Figure 4, i.e. human islets were pre-exposed to Imatinib for 6 hours and then cultured for another 24 hours with or without Imatinib and hydrogen peroxide, streptozotocin and DETA/NO (Figure 5), or with or without Imatinib and cytokines (Figure 6). We observed that islet release of the chemokine IL-8 to the culture medium was increased by Imatinib (Figure 5A). This observation corroborates the Imatinib-induced expression of IL-8 mRNA assessed by microarray analysis (Table 1). The beta-cell cytotoxic agents hydrogen peroxide, streptozotocin and DETA/NO did not increase human islet release of IL-8 (Figure 5A), which is in agreement with our observation that IκB-α and STAT1 phosphorylation were not affected by these toxins (Figure 4). Imatinib increased the release of IL-8 also in islet cells exposed to hydrogen peroxide and DETA/NO (Figure 5A), supporting the notion that Imatinib enhances basal NF-κB activity and chemokine release. Due to large error bars, there were no significant effects of Imatinib and the cytotoxic agents on IP10 (Figure 5B) and MCP-1 (Figure 5C) release. Nevertheless, there was a trend to higher IP10 and MCP-1 release in response to Imatinib, also supporting an NF-κB stimulatory effect of Imatinib. The levels of MIG and RANTES were barely detectable and we could not observe any effects on the release of these chemokines (results not shown).

The release of IL-8, IP10, MIG and MCP-1, but not of RANTES, was dramatically (100–1000-fold) increased by the cytokines IL-1ß and IFN-γ (Figure 6). Interestingly, Imatinib decreased the cytokine-induced release of IP10 and MIG, and tended to decrease MCP-1 and IL-8 release (Figure 6). Thus, a prolonged Imatinib exposure decreased both cytokine-induced transcription factor phosphorylation (Figure 4) and chemokine release. To validate the results obtained from the microarray and the chemokine release kit, we analyzed IL-8 mRNA levels in human islets using real time PCR. Imatinib exposure for 30 hours resulted in a 8-fold induction of IL-8 mRNA levels when compared to control. (Figure 6 B). Cytokine (IL-1ß and IFN-γ) treatment for 30 hours led to a strong induction (over a 35-fold) of IL-8 mRNA levels. (Figure 6 B). However when pretreated with Imatinib, the cytokine-induced IL-8 mRNA expression was significantly inhibited (Figure 6 B). The result from real time PCR are in concert with the chemokine release and microarray data, further strengthening the notion that a prolonged Imatinib exposure dampens the cytokine-induced chemokine release in human islets.

### Prolonged Imatinib exposure leads to decreased κB-binding in in INS-1 832/13 cells

**Figure 7:**
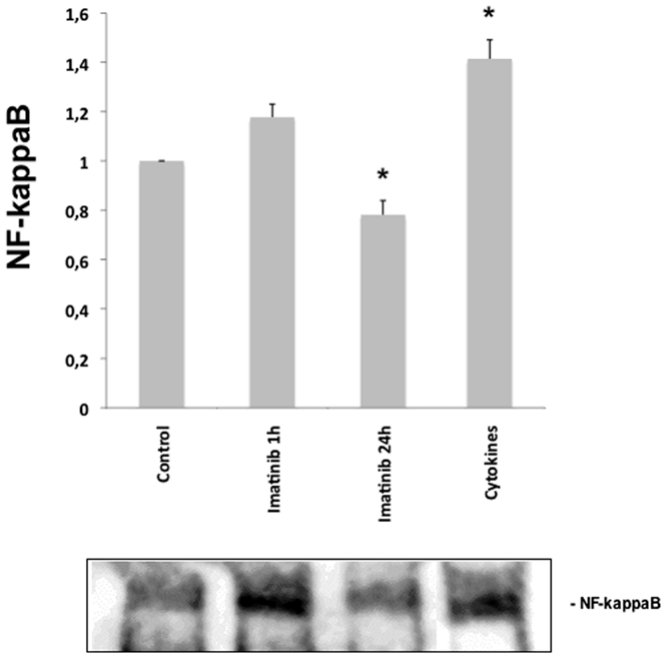
Effects of Imatinib on κB-binding activity in INS-1 nuclear extract. INS-1 832/13 cells were left untreated (control), treated with cytokines (50 U/ml IL-1β+1000 U/ml IFN-γ for 30 min or treated with 10 µM Imatinib for 1 or 24 hours. Nuclear extracts were prepared and NF-κB activity was determined by EMSA. The upper panel shows densitometric scanning results ± SEM for 3 separate experiments. * denotes p<0.05 versus control using Student's t-test.

Human islets consist of 30–60% beta-cells. However they also contain other endocrine cells such as alpha- and delta-cells. To confirm that the observed effects of Imatinib on NF-κB activation in human islets are specific for beta-cells we performed electromobility shift assay (EMSA) on nuclear extracts from the INS-1 832/13 beta-cell line. Cytokine (IL-1β and IFN-γ) exposure for 20 min resulted in a significant increase in κB-binding activity in INS-1 832/13 nuclear extracts (Figure 7). A short time Imatinib exposure (1 hour) tended to increase the κB-binding activity (p = 0.06) whereas a long exposure (24 hours) of INS-1 cells to Imatinib resulted in a significant decrease of κB-binding activity (Figure 7). These results strengthen the idea that a short-term exposure to Imatinib results in a modest activation of NF-κB, whereas a prolonged Imatinib exposure dampens NF-κB activation in beta-cells.

## Discussion

We presently report that both Imatinib and the cytokines IL-1β and IFN-γ increase IκB-α phosphorylation, ser276-p65 phosphorylation and lys221-p65 methylation. The cytokine-induced phosphorylation of IκB-α in insulin producing cells has previously been reported [3]–[5], but the methylation event is, to our knowledge, a novel finding. The methylation of p65 at position lys221 appears to be a necessary event for mouse embryo fibroblast p65-dependent gene expression, and may be carried out by a lysine methylase, the nuclear receptor–binding SET domain-containing protein 1 (NSD1) [33]. The demetylation is probable carried out by the lysine demethylase F-box and leucine-rich repeat protein 11 (FBXL11), a protein that is induced by NF-κB and therefore functions as a negative feedback mechanism [33]. The present findings indicate that this NF-κB regulatory mechanism is probably operational in human islet cells and that both cytokines and Imatinib stimulate NF-κB activity by enhancing p65 methylation. Although the mechanisms underlying increased p65 methylation remain to be elucidated, immunoblot assessment of p65 methylation could provide a convenient and accurate alternative to other technique currently used for the detection of islet NF-κB activation.

Also the increased phosphorylation of p65 at position ser276 points to an NF-κB stimulating effect of Imatinib. A previous publication has reported that IL-1β -induced NF-κB activity in insulin-producing cells requires p65 phosphorylation at position ser276, but not at ser536 and ser529 [34]. This is in agreement with our present finding that both cytokines and Imatinib stimulated ser276, but not ser536 phosphorylation. Ser536 phosphorylation may require the PI3K/PKB pathway [35], whereas ser276 phosphorylation of p65 may result from activation of PKA [36], MSK1 [37] or Pim-1 [38]. We have previously observed that cytokines activate the p38MAPK/MSK1 pathway in insulin producing cells [39], indicating that cytokine-induced p65 phosphorylation involves p38MAPK/MSK1 activity rather than PI3K/PKB. P38MAPK has also been reported to promote p300 association with p65 and the subsequent acetylation of p65 at position lys310 [40]. Although we did not observe p65 acetylation in human islets, we cannot exclude the possibility that this occurs, and we hope that the future availability of a more sensitive p65 acetylation antibody might resolve this question.

We verified Imatinib-induced activation of human islet NF-κB by microarray, real-time PCR and measurement of chemokine release. These approaches identified the chemokine IL-8, which is a well-known NF-κB target gene in islet cells [41], to be induced by Imatinib. However, the Imatinib-induced increase in IL-8 was very modest - 2-fold - as compared to the effect of cytokines, which was almost 1000-fold. This may indicate that other NF-κB synergizing signaling pathways are activated by the cytokines IL-1β and IFN-γ, for example IRF-1 and STAT1, and that the effect of Imatinib is too weak to promote clinical inflammation.

Of greater functional importance appears to be the Imatinib-induced decrease in cytokine-induced chemokine release. Cyto/Chemokines can exert both beneficial and detrimental effects on islets. For example, short time exposure of islets to IL-1β promotes beta-cell replication and insulin secretion whereas exposure for longer time periods leads to beta-cell death [42]. Islet cells, including beta-cells, produce a variety of chemokines that are necessary for maintaining beta-cell function and mass. However, if the cytokine balance is disturbed, an inflammatory response within the islet can damage or in the worst case destroy the islet. We report that Imatinib significantly decreased the cytokine-induced release of the chemokines MIG and IP10 and, although not reaching statistical significance, tended to dampen the release of IL-8. IP10, MIG and IL-8 belong to the CXC family of chemokines and increased levels have been associated with islet inflammation and Type 1 and Type 2 diabetes in both animal models and in humans [43]. One could therefore speculate that the observed antidiabetic effects of Imatinib in vivo in part could be due to a lowered islet secretion of the above chemokines. Although the effects of Imatinib NF-κB activation were validated using a beta-cell line, we cannot exclude that Imatinib modulates NF-κB activity in other islet cells in a similar way.

**Figure 8:**
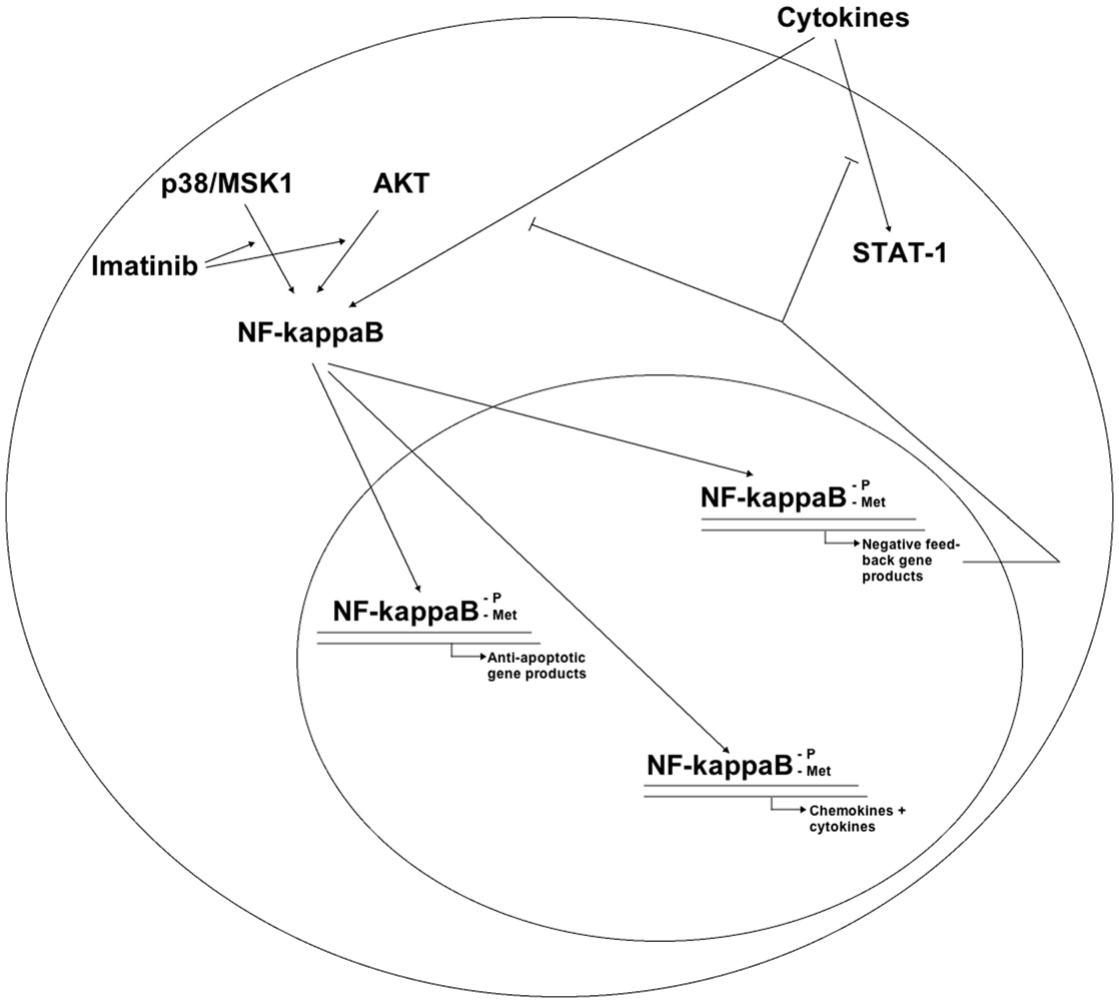
Hypothetical model for Imatinib-induced modulation of NF-κB activation in human islet cells. The results of the present investigation are compatible with the view that Imatinib, possibly via inhibition of c-Abl and stimulation of MSK1 and AKT, activates NF-κB, as assessed by p65 serine phosphorylation and methylation. Activated NF-κB stimulates the expression of anti-apoptotic genes, cyto/chemokine genes (IL-8, MIG, IP10) and negative feedback genes (IκB, I-TRAF, IL4R). The negative feedback genes will desensitize islet cells to cytokines so that a subsequent cytokine exposure results in a dampened NF-κB and STAT-1 response.

Our results point to the possibility that the Imatinib-mediated increase in basal NF-κB activity stimulates NF-κB-induced negative feedback signals that dampen the cytokine-mediated inflammatory response (Figure 8). Examples of such negative feedback mechanisms are the above-mentioned FBXL11 demethylase [33], NF-κB -induced IκB-gene transcription, IκK-β -induced A20 phosphorylation [44], [45], SOCS3 [46] and ABIN1-3 [47]. In addition, the scaffold protein I-TRAF has recently been reported to inhibit NF-κB activation by recruiting polo-like kinase 1 [48], and IL-4R-mediated STAT6 activation has been shown to prevent p65 nuclear translocation [49]. Interestingly, we presently observed induction of I-TRAF and IL-4R mRNA in response to Imatinib. Thus, it is tempting to speculate that a long-term Imatinib exposure enhances I-TRAF and IL-4R expression, which will in the long term negatively regulate cytokine-induced NF-κB activation.

The precise molecular targets for Imatinib that mediate modulation of NF-κB activity in human islet cells are unknown. Imatinib is known to inhibit the tyrosine kinases c-Abl, PDGFR, c-Kit and DDR1/2. Among these c-Abl may be of particular interest because it is known that c-Abl suppresses basal NF-κB activity in mouse embryonic fibroblasts [50]. C-Abl seems to promote both tyrosine phosphorylation of IκB-α and downregulation of the histone deacetylase HDAC1, a negative regulator of NF-κB [50]. It has therefore been suggested that the proapoptotic activity of c-Abl may be mediated by downregulation of NF-κB activity [50]. The findings of this study suggest that not only c-Abl mediated apoptosis [24], but also c-Abl control of chemokine production is targeted by Imatinib treatment.

In summary, we presently observe that Imatinib in human islets, possibly via inhibition of c-Abl, initially promotes an increased basal NF-κB activity, which is followed by, somewhat paradoxically, a second stage characterized by a lowering in the sensitivity to cytokines. This may result in two beneficial effects in diabetes: Firstly, induction of anti-apoptotic genes will probably stimulate beta-cell survival and insulin production. Secondly, lowered cytokine sensitivity might decrease islet leukocyte infiltration, amyloid formation and beta-cell exhaustion. Both effects may contribute to the observed anti-diabetes effects of Imatinib in humans and in animal models for Type 1 and Type 2 diabetes.
